# Evaluation of hydrocephalus and other cerebrospinal fluid disorders with MRI: An update

**DOI:** 10.1007/s13244-014-0333-5

**Published:** 2014-06-06

**Authors:** Merve Gulbiz Kartal, Oktay Algin

**Affiliations:** Department of Radiology, Ataturk Training and Research Hospital, 06050 Bilkent, Ankara Turkey

**Keywords:** MRI, Hydrocephalus, CSF, 3D-SPACE, Endoscopic third ventriculostomy, Ventriculoperitoneal shunt

## Abstract

MRI is not only beneficial in the diagnosis of cerebrospinal fluid (CSF)-related diseases, but also aids in planning the management and post-surgery follow-up of the patients. With recent advances in MRI systems, there are many newly developed sequences and techniques that rapidly enable evaluation of CSF-related disorders with greater accuracy. For a better assessment of this group of disorders, radiologists should follow the developments closely and should be able to apply them when necessary. In this pictorial review, the role of MRI in the evaluation of hydrocephalus, CSF diversion techniques, and other CSF disorders is illustrated.

*Teaching Points*

• *The 3D-SPACE seems to be most efficient technique for evaluation of hydrocephalus and ventriculostomy.*

• *In complex cases, PC-MRI, 3D-heavily T2W, and/or CE-MRC images may prevent false results of 3D-SPACE*.

• *MRI is beneficial in the diagnosis and management of hydrocephalus and other CSF-related diseases.*

## Introduction

Progressive developments in magnetic resonance imaging (MRI) technologies allow us to better assess CSF circulation. Therefore, MRI aids in the diagnosis of diseases that result from alterations of the CSF circulation. Hydrocephalus, which constitutes a major CSF-related disorder, is well demonstrated using MRI. MRI also helps to discriminate the aetiology of the disease [1]. The provided data are important for planning the management as well as follow-up of the patients. MRI is also effective in the diagnosis and treatment planning of other CSF disorders such as CSF leakage, arachnoid cysts, etc. [2–4]. In this review, the role of MRI in the evaluation of hydrocephalus and other CSF disorders with emphasis on the most recently used sequences in routine practice is covered.

## CSF circulation

The CSF volume is approximately 150 ml in adults; 125 ml is distributed in the cranial and spinal subarachnoid spaces and 25 ml is found in the ventricles [1]. A volume of 400–500 ml is secreted and approximately 330–380 ml of CSF enters the venous circulation daily [2].

CSF is produced in the choroid plexus, brain parenchyma, spinal cord, and ependymal lining of the ventricles. Most is secreted in the lateral ventricles and leaves the ventricles through the foramen of Monro to enter the third ventricle. From there, the CSF flows into the fourth ventricle through the aqueduct. It leaves the fourth ventricle by the foramen of Magendie and foramina of Luschka and enters the subarachnoid space. Cerebrospinal fluid is essentially absorbed into the internal jugular system via cranial arachnoid granulations. However, multiple experiments indicate that movement along nerve roots and exiting vessels also plays a role [1, 3]. In addition, absorption towards the interstitial compartment occurs via the Virchow-Robin spaces [1].

## Hydrocephalus

Hydrocephalus is a complex disorder that can develop for various reasons. Dilatation of the ventricular system may lead to loss of brain cells resulting in a variety of neurological symptoms, stroke, and sometimes even death due to pressure applied on the brain parenchyma [4]. The causes of CSF increase are often obstructive diseases such as cystic lesions, tumours or obstructive membranes [5–7]. Rarely, it may be the result of excessive CSF production, which may be due to pathologies at the sites where CSF production takes place. More frequently, it is be due to an obstruction in the ventricular system (obstructive or non-communicating type) or interrupted CSF absorption or flow (communicating type) [8]. In young adults and children, obstructive-type hydrocephalus is the most common type [6, 9, 10]. In some instances, such as meningitis, both absorption and flow may be interrupted, which is defined as complex-type hydrocephalus [11]. Although there are several theories regarding the pathophysiology of hydrocephalus, recently the most widely accepted one has been Greitz’s hyperdynamic flow theory, which divides hydrocephalus into two main groups, acute and hydrocephalus [8]. Acute hydrocephalus is caused by an intraventricular CSF obstruction. Chronic hydrocephalus is further divided into communicating and chronic obstructive hydrocephalus. The theory proposes that chronic hydrocephalus is a result of decreased intracranial capillaries, which causes restricted arterial pulsations and increased capillary pulsations and decreased intracranial compliance [8, 9].

**Fig. 1:**
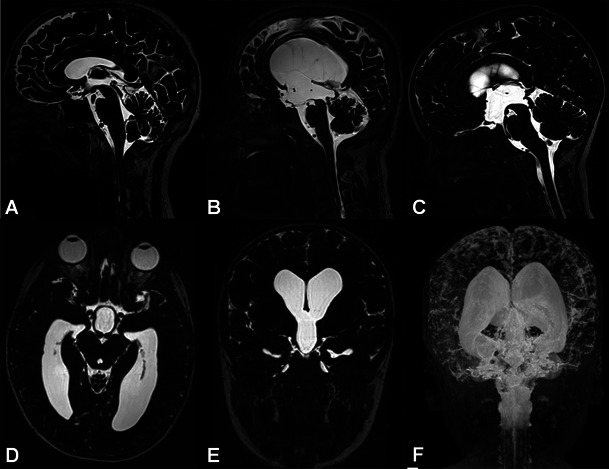
Heavily T2W 3D-SPACE images of different cases. A normal midline sagittal image is shown for comparison (**A**). The rest of the images from two different patients with aqueductal stenosis demonstrate enlargement of the ventricles proximal to the obstruction (**B**, **C**), enlargement of the third ventricular recesses (**B**, **C**), dilated ventricular horns (**D**–**F**) and narrowed cortical sulci (MIP image, **E**), which are typical findings of obstructive hydrocephalus

**Fig. 2:**
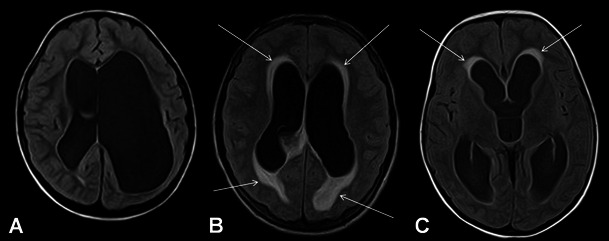
Axial FLAIR images of three different patients with hydrocephalus. In the first patient with chronic compensated hydrocephalus, lack of periventricular CSF resorption is seen (**A**). In the second patient, periventricular hyperintensity consistent with interstitial oedema due to acute decompansated hydrocephalus is demonstrated (*arrow*, **B**). Periventricular caps seen in middle aged adults should be differentiated from decompansated hydrocephalus (*arrows*, **C**)

The most commonly used radiological criteria in the diagnosis of hydrocephalus are given below [12, 13] (Figs. 1 and 2);

1. Ventriculomegaly (Evans' index >0.3),
2. Enlargement of the third ventricular recesses and lateral ventricular horns,
3. Decreased mamillopontine distance and frontal horn angle,
4. Thinning and elevation of the corpus callosum,
5. Normal or narrowed cortical sulci,
6. Periventricular white matter hyperintensities (interstitial oedema and acute hydrocephalus),
7. Aqueductal flow void phenomenon in T2W images (a sign of communicating hydrocephalus).

These criteria are not specific for hydrocephalus, and their sensitivities are poor [13]. The gold standard diagnostic method for hydrocephalus is ventriculographic studies [6]. On the other hand, this is a highly invasive method and may lead to serious complications. Therefore, new MRI techniques have been developed in order to determine the aetiology and treatment. These techniques include phase-contrast MRI (PC-MRI), three-dimensional (3D) heavily T2W sequences and contrast-material-enhanced MR cisternography (CE-MRC) [14, 15]. All three techniques have their own advantages and disadvantages.

PC-MRI provides quantitative and qualitative data regarding CSF circulation. On the other hand, in the presence of complex or turbulent flow, results may be false positive or negative [14, 15]. Another drawback of the technique is that it is extremely sensitive to technical factors [16]; 3D heavily T2W sequences [such as 3D-DRIVE (Philips), 3D-CISS (Siemens) or FIESTA-C (GE)] may provide accurate anatomical data. However, these techniques lack physiological information [5–7]. CE-MRC is an invasive test and is highly dependent on radiologist experience [6].

In recent years, 3D sampling perfection with application-optimised contrast using the variable flip-angle evolution (3D-SPACE) technique has been developed and shown to be useful in the evaluation of patients with obstructive hydrocephalus [5]. The technique allows scanning of the whole cranium in an acceptable acquisition time using isotropic voxels (with voxel size < 1 mm^3^), which is useful in obtaining high-resolution multiplanar reformatted images without exceeding specific absorption rate (SAR) limits. Other advantages are that the technique is a flexible one allowing employment of different sequence types such as T1W, T2W, fluid-attenuated inversion-recovery (FLAIR), proton-density weighted or variant flip-angle mode T2W images. Also, the 3D-SPACE technique is non-invasive and less sensitive to artefacts [17–19]. Our 3-T MRI protocol for patients with hydrocephalus is given in Table 1.

**Table 1:** Our 3-T MRI protocol for patients with hydrocephalus. PC-MRI and 3D-heavily T2W images are optional

	3D-MPRAGE	3D-SPACE	PC-MRI (Qualitative)	3D-heavily T2W (3D-SPACE)	PC-MRI (Quantitative)
TR/TE (ms)	2,130/3.45	3,000/579	34.9/9.8	3,000/526	30/7.43
TI (ms)	1,100	-	-	-	-
Slice thickness	0.8 mm	0.6 mm	4 mm	0.7 mm	4 mm
FOV* (mm)	230 × 230	240 × 240	240 × 240	240 × 240	240 × 240
Acquisition time	5.5 min	6 min	5 min	5 min	5 min
Velocity encoding	-	-	6 cm/s	-	20 cm/s
NEX	1	2	2	2	1
Number of slices	240	240	1	240	1
Flip angle	8°	Variable	10°	120°	10°
Imaging plane	Sagittal	Sagittal	Axial-sagittal	Sagittal	Axial
Distance factor	50 %	-	-	-	-
PAT factor	2	2	None	2	None
PAT mode	GRAPPA	GRAPPA	-	GRAPPA	-
Voxel size (mm)	0.8 × 0.8 × 0.8	0.6 × 0.6 × 0.6	-	0.7 × 0.7 × 0.7	-
FA mode	-	T2 variant	-	T2 constant	-

Note: TI: time of inversion; 3D-SPACE: three-dimensional sampling perfection with application optimised contrasts using different flip angle evolutions; 3D-MPRAGE: three-dimensional T1W magnetisation prepared rapid acquisition gradient-echo; PC-MRI: phase-contrast cine MRI; NEX: number of excitations; FOV: field of view; PAT: parallel acquisition technique; GRAPPA: generalised auto calibrating partially parallel acquisitions

Below imaging findings in different types of hydrocephalus have been summarised.

### A. Non-communicating (obstructive) hydrocephalus

Similar pathologies may lead to obstruction in different locations. The location, aetiology and severity of obstruction are crucial in order to plan the treatment procedure [4]. It should be taken into consideration that any space-occupying lesion with significant size that compresses the foramina from outside or any intraventricular lesions and haemorrhage may lead to obstructive hydrocephalus [6, 7, 9]. Acute hydrocephalus is an emergency condition that should be treated urgently. Unless the condition is urgently treated, it may lead to serious complications including persistent blindness, cerebral infarct, herniation or death [20].

The most significant finding on MRI to discriminate between acute and chronic forms of hydrocephalus is periventricular hyperintensities on T2W or FLAIR images, which is consistent with acute interstitial oedema [10] (Fig. 2). Below, the obstruction sited are evaluated regarding pathologies specific to that site and MRI.

#### Foramen of Monro

**Fig. 3:**
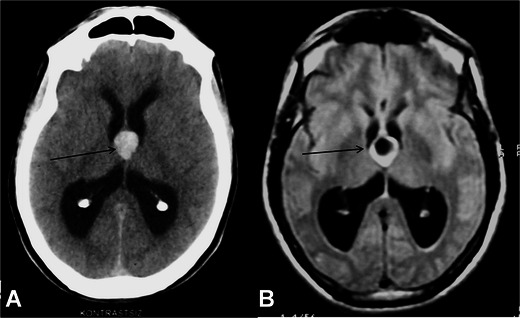
A 46-year-old male patient with a colloid cyst. Axial noncontrast-material enhanced CT image demonstrates hyperdense lesions located in the foramen of Monro sized 2 cm in diameter (*arrow*, **A**). The lesion is hypointense in FLAIR images, which indicates the proteinous content of the cyst (*arrow*, **B**)

Unlike the other sites of obstruction, obstruction of the foramen of Monro may lead to unilateral ventriculomegaly [4]. Tumours arising from adjacent tissues such as glial tumours, epandimomas and subepandymomas, intra- or periventricular cysts such as arachnoid, dermoid or epidermoid cysts, adhesions due to previous haemorrhage or ventriculitis may lead to hydrocephalus by obstructing the foramina [4, 9]. The lesion that causes obstruction specifically in this particular area is the colloid cyst [21]. Although there may be variations in signal characteristics on MRI depending on the content of the cyst, it generally appears as a hyperintense lesion on T1W images and may be iso- to hyperintense on T2W images. Cysts with proteinaceous debris may show hypointensity on T2W or FLAIR images (Fig. 3). It is harder to aspirate these kinds of cysts [20]. They may require a shunt procedure or total excision.

#### Aqueductus cerebri (aqueduct)

**Fig. 4:**
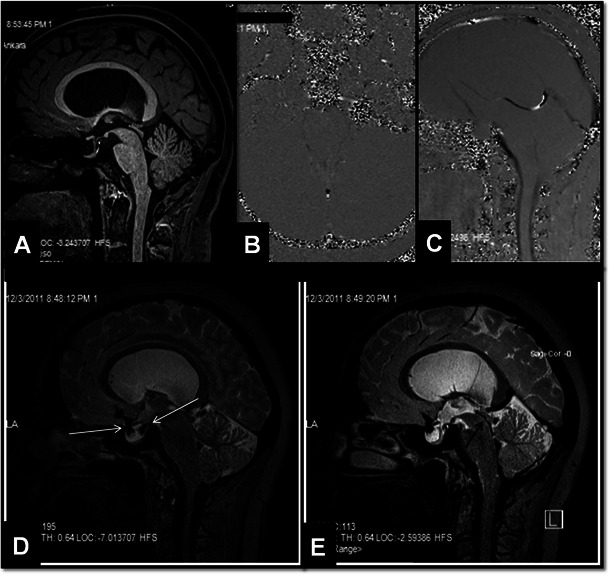
A 32-year-old female patient with partial aqueductal stenosis, partial empty sella and hydrocephalus. In the sagittal midline 3D-MPRAGE image no increase in third ventricle size and no aqueductal abnormalities are detected (**A**). However, axial and sagittal PC-MRI images (VENC value: 6 cm/s) demonstrate lack of CSF flow in the aqueduct (**B**, **C**). Sagittal 3D-SPACE image demonstrates passage of CSF from basal-prepontine cisterns into the sellar cavity through the diaphragm sella (*white arrows*, **D**). This finding explains why hydrocephalus may be associated with empty sella in most of the patients. In the midline sagittal 3D-SPACE image, a narrowed but patent aqueduct is demonstrated (*black arrow*, **E**)

Aqueductal stenosis (AS) can be classified as congenital or acquired [14, 17]. The most common lesions causing AS are pineal gland tumours, gliomas in the tectum-tegmentum, tentorial meningiomas, Galen vein aneurysm, web, synechia and cysticercosis [4, 6]. Generally, advanced MRI techniques are needed in order to diagnose and determine the aetiology of the stenosis. PC-MRI aids in evaluating aqueductal patency [14]. Axial and sagittal images are beneficial when performing PC-MRI. Axial plane images encode in the craniocaudal direction for flow quantification and sagittal plane images encode craniocaudal images for qualitative evaluation. Quantitative CSF velocity and qualitative flow information can be obtained in an additional 8–10 min in connection with routine MRI. The aqueductal web may be accurately visualised on 3D heavily T2W images [12]. MRC provides additive data in controversial cases [6]. These sequences have also been reported to be useful in the assessment of the treatment response [4, 14]. Recent studies have shown that 3D-SPACE with the variant flip angle mode (VFAM) technique alone is usually sufficient for the diagnosis of AS by itself [17] (Fig. 4). Furthermore, a heavily T2W 3D-SPACE sequence with constant flip-angle images is beneficial in demonstrating luminal morphology [17, 19]. The absence of hypointense signal from the third ventricle into the fourth ventricle (also called the flow void sign) from CSF on 3D-SPACE with variant flip-angle mode images indicates aqueduct stenosis. Compared to the other fully balanced techniques, such as 3D-CISS and other flow-compensated gradient echo sequences, the 3D-SPACE technique is less sensitive to artefacts while providing images with similar contrast and geometric resolution [17–19].

#### Fourth ventricular outlet (FVO)

**Fig. 5:**
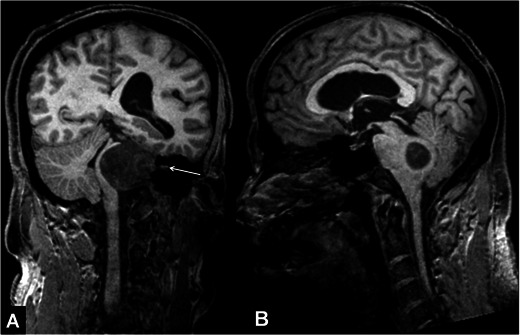
Reformatted coronal (**A**) and sagittal (**B**) 3D-MPRAGE images of a 50-year-old male patient with a left cerebello-pontine angle mass and hydrocephalus. Coronal image demonstrates the mass extending out of the internal acoustic canal, decompressing and displacing midbrain structures to the right (*arrow*, **A**). In sagittal images it is shown that the mass narrows the fourth ventricle and fourth ventricular outlet (**B**)

Unfortunately, there has been no systematic research or case series to evaluat the FVO. The literature is limited to case reports [12]. Heavily T2W 3D sequences or the 3D-SPACE technique with VFAM may be used in order to detect obstructions [4]. On the other hand, CE-MRC is usually helpful in equivocal cases [6]. The most common reasons that lead to obstruction of the foramina of Luschka and Magendie are bleeding, meningitis and extrinsically decompressing lesions such as neoplasms and cranio-cervical developmental malformations [4] (Fig. 5). Among the posterior fossa neoplasms the most commonly seen lesions in paediatric age patients are medulloblastomas, cerebellar astrocytomas and brain stem gliomas [4, 9]. Rarely, posterior circulation infarctions may lead to FVO obstruction and acute hydrocephalus due to mass effect.

#### Foramen magnum

**Fig. 6:**
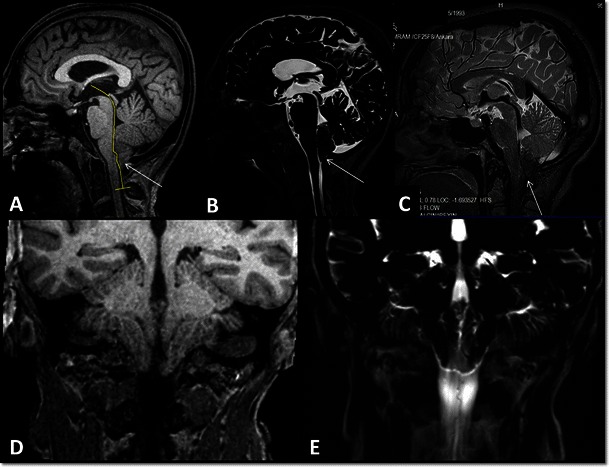
A 19-year=old female patient with Chiari malformation. Sagittal 3D-MPRAGE (**A**) and heavily T2W 3D-SPACE (**B**) images demonstrate cerebellar tonsils extending into the foramen magnum (*arrows*). 3D-SPACE with variant FA mode image shows the narrow foramen of magendi and foramen magnum (*arrow*, **C**). Thinned hypointense signal in these foramina is due to decreased CSF flow (*arrow*, **C**). On coronal curved reformatted 3D-MPRAGE (**D**) and heavily T2W 3D-SPACE (**E**) images, the patency of the foramina and position of the cerebellar tonsils are better evaluated. The coronal curved reformatted images are obtained by drawing a line on sagittal images as in Fig. 6a (yellow line in A)

Conditions that may lead to narrowing of the foramen magnum include osteochondrodysplasias, metabolic disorders, developmental abnormalities and Chiari malformations [12]. A narrowed foramen magnum may often be followed by intracranial hypertension due to interruption of cerebral venous return at the level of the jugular foramen [12, 22]. In these cases, although the techniques explained above are beneficial, we suggest implementing a 3D T1W sequence in that MRI protocol that may be 3D T1W [magnetisation-prepared 180 degree radio-frequency pulses and rapid gradient-echo (MPRAGE)] in addition to the T2W (3D-SPACE) sequences for an optimum morphological analysis (Fig. 6). Although PC-MRI has been shown to be useful in the evaluation of CSF circulation at the level of the posterior fossa and foramen magnum, the need for this technique has gradually decreased with the use of 3D-SPACE with the VFAM technique [16].

### Communicating Hydrocephalus

**Fig. 7:**
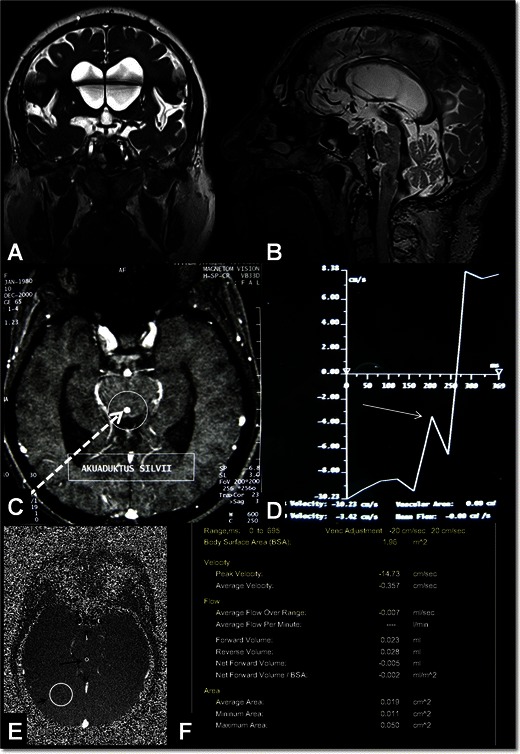
A 62-year-old male with normal pressure hydrocephalus (NPH). Coronal TruFisp image shows enlarged Sylvian cisterns, tight medial parietal sulci and ventriculomegaly (**A**). Sagittal 3D-SPACE image shows hypointense hyperdynamic CSF flow in the aqueduct and fourth ventricle (**B**). Axial PC-MRI examination at another centre shows aqueductal ROI placement (broken arrow) for determination of CSF flow (**C**). Aliasing seen in the flow chart occurred because the VENC value was selected as less than what it should be (VENC: 10 cm/s) (arrow, **D**). On axial phase images obtained in the PC-MRI examination carried out by selecting a Venc value of 20 cm/s, ROIs on the aqueduct (long arrow) and right occipital lobe (reference ROI, short arrow) are seen (**E**). Maximum aquaductal CSF flow is calculated as 14.73 cm and stroke volume was calculated as 0.051 ml (**F**)

The characteristic example in this group is normal pressure hydrocephalus (NPH), which manifests with gait disturbance, urinary incontinence and dementia [13, 23, 24]. Early and accurate diagnosis is critical in the management of NPH since among all the conditions that cause dementia, NPH, which is treated by CSF diversion, is the only one that can be treated [23–25]. On the other hand, response to CSF diversion treatment is 50-60 % [26]. It was reported that patients diagnosed and treated in the early stage respond better to treatment [24, 26]. Although conventional MRI sequences may reveal morphological findings, certain diagnoses require demonstration of hyperdynamic aqueductal flow on PC-MRI [23, 27] (Fig. 7). PC-MRI parameters are useful for the diagnosis, but they are not sufficient to predict treatment response [23, 27]. Recent studies have demonstrated that CT cisternography or CE-MRC provides additive data for the diagnosis of NPH and predicting treatment response [6, 26, 28]. On the other hand, today there are no methods to predict treatment response with high sensitivity, specificity and accuracy [24–28].

### Evaluation of treatment procedures (third ventricle integrity, patency of ventriculostomy and ventriculo-peritoneal shunt)

Although the causes of hydrocephalus vary in a wide range, treatment procedures are basically alike (ventriculostomy or ventriculo-peritoneal shunt). It is still debated which group of patients would benefit more from which treatment. Today, mainly two methods are used in the treatment of hydrocephalus. The first one is the ventriculoperitoneal shunt (VPS) including treatment with catheters that are placed proximal to the obstruction to take out excess CSF. Mortality, morbidity and complication rates are high for VPS and 50 % of patients face various problems within 2 years [4, 6, 9]. Thus, shunt revision is generally required for most of the patients. The second one [endoscopic third ventriculostomy (ETV)] is described as entering into the brain with an endoscopic procedure and opening a new drainage pathway that bypasses the obstruction inside of the brain. ETV is a more physiological treatment than VPS. The most important complication with this method is bleeding due to injury to the basilar artery or one of its branches that courses near the surgery site, and this may lead to death.

The CSF diversion procedures explained above are carried out according to previously determined anatomic landmarks without visualising the whole brain and intracranial structures. In some of the patients with hydrocephalus, deformation and displacement or asymmetric enlargement of ventricles can be seen, increasing the failure and complication rates in these procedures. This makes preoperative evaluation critical in patients who will undergo ETV and VPS procedures (especially ETV). The preoperative ETV evaluation should include detailed assessment of landmarks that the endoscope will pass through such as the ventricular system, integrity of the third ventricle, patency of the Liliequist membrane, and positions of basilar artery-mammillary bodies and basal cisterns [12, 29]. In the presence of spontaneous third ventriculostomy (STV), ETV is unnecessary. Therefore, it should be investigated in the preoperative period [6, 15, 30]. T2W 3D-SPACE with the VFAM technique is usually sufficient for the preoperative evaluation [5, 18].

**Fig. 8:**
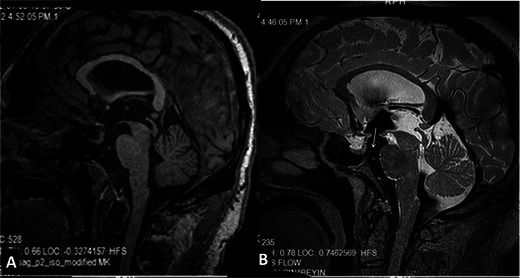
Sagittal 3D-MPRAGE (**A**) and 3D-SPACE with variant FA mode (**B**) images of a 41-year-old male patient with aqueduct stenosis and a history of previous ETV. 3D-SPACE image demonstrates that the ETV stoma is patent and CSF flow is clearly seen (*arrow*, B)

After successful ETV, expected findings on routine MRI include regression in ventricle size, interstitial oedema and demonstration of the flow void sign in the stoma [12, 29]. Flow in the stoma should be demonstrated while evaluating the images for ETV patency [16]. All these findings are demonstrated effectively with T2W 3D-SPACE with the VFAM technique (with isotropic voxels and voxel size < 1 mm^3^) within an acquisition time of approximately 5 min [18] (Fig. 8). In controversial cases heavily 3D T2W, thin-section T2W TSE, PC-MRI and/or MRC images can be used as adjunctive techniques [4, 5]. On the other hand, correlation with clinical findings and close follow-up are critical since the failure rate is 40 % on long-term follow-up and sudden death has been reported [13].

**Fig. 9:**
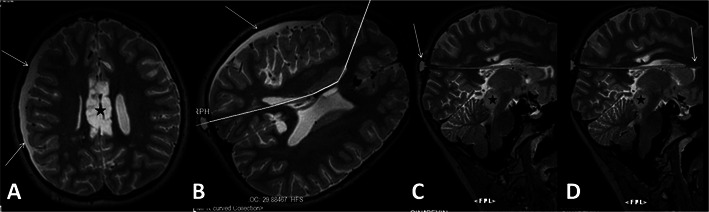
Reformatted 3D-SPACE images of an 11-year-old girl with aqueduct stenosis and hydrocephalus. Axial images show right temporal-parietal subdural haematoma (arrows in **A** and **B**) and iatrogenic callosal injury (asterisk in **A**). On reformatted sagittal 3D-SPACE images obtained at the level of the shunt catheter, as shown in Fig. 6B, the position and integrity of the catheter and shunt reservoir can be precisely evaluated (*arrows* in **C** and **D**). Distal end of the catheter is shown to be in the corpus callosum (*arrow* in **D**). Also, tectal glioma is well demarcated in sagittal images (*asterisks* in **C** and **D**)

Also, MRI is a useful method for evaluating VPS. Position and integrity of the VPS catheter and other complications (e.g. subdural effusions, damage to neuronal tissues) can be easily evaluated by the 3D-SPACE technique with small isotropic voxels (Fig. 9). PC-MRI and/or 3D-SPACE with VFAM techniques can also be used to assess VPS patency [16].

## The role of MRI in other CSF disorders

### Arachnoid cysts (ACs)

**Fig. 10:**
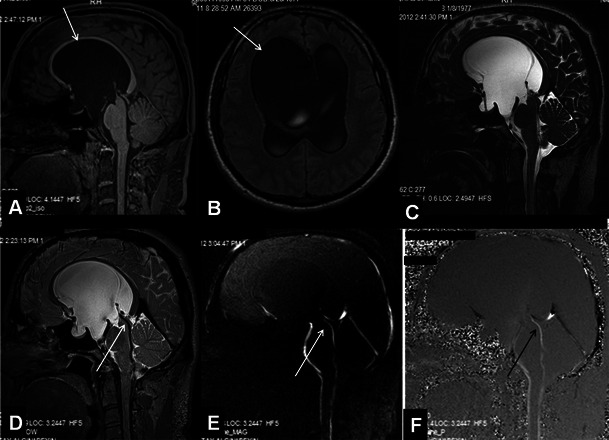
A 34-year-old male patient with intraventicular arachnoid cyst (AC) and hydrocephalus. Sagittal 3D-MPRAGE (**A**) and axial FLAIR (**B**) images show AC extending from the right lateral ventricle into the third ventricle (arrows). Sagittal heavily T2W 3D-SPACE image clearly shows the morphology of the AC and third ventricle (**C**). Sagittal 3D-SPACE with variant FA mode (**D**) and PC-MRI (**E, F**) images demonstrate the narrowed aqueduct and decreased aqueductal CSF flow (*arrows*)

Arachnoid cysts are mostly found in the temporal fossa and cerebellopontine angle, but they may also be located intra- or periventricularly [31]. Due to CSF secreted from the cyst wall or check-valve mechanism, which allows inflow of CSF but prevents outflow, these lesions may grow larger and compress ventricles and foramina [6, 9, 31] (Fig. 10). ACs appear as homogeneous cystic lesions with smooth margins, isointense to CSF in all sequences [21]. Conventional cranial MRI sequences usually enable diagnosis of AC, but for those lesions that are controversial, diffusion-weighted and post-contrast T1W images are beneficial [6, 21]. Demonstrating the communication of ACs with the ventricular and subarachnoid system is essential in order to plan the treatment. PC-MRI or CE-MRC may be useful to identify the presence of the communication [6, 31].

### Rhinorrhoea and otorrhoea

**Fig. 11:**
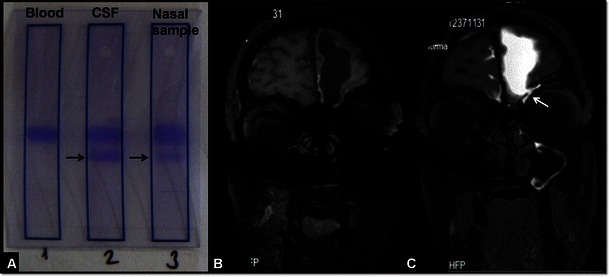
A 32-year-old male patient with rhinorrhoea and a history of trauma. Electrophoresis study (**A**) shows no beta-2-transferrin in the blood sample assigned as no. 1, whereas in the columns assigned as 2 (CSF) and 3 (nasal sample) beta-2-transferrin is demonstrated (arrows). Coronal precontrast fat-saturated T1W images cannot precisely locate the level of CSF leakage (**B**). After intrathecal Gd-DTPA administration, postcontrast T1W image obtained with the same parameters clearly demonstrates the leakage into the left frontal sinus (*arrow*, C)

CSF leakage from the nose is defined as rhinorrhoea, while leakage from the ear is called otorrhoea. CSF leakages may be traumatic, non-traumatic or spontaneous in origin, but the evaluation algorithm is the same for all three subtypes [32]. In all patients the first step is identifying the presence of β-2 transferrin in order to make a certain diagnosis (Fig. 11) [33]. Thin-section CT images are beneficial in the evaluation of traumatic CSF leakages. However, CT is usually inadequate in cases with multiple fractures and intermittent-low flow fistulas [6]. CT or radionuclide cisternography are not sufficient for low-flow or hair-like leakages either [6, 33]. The most important drawback of these methods is their invasive nature and radiation exposure [34]. These limitations are overcome in non-contrast MR cisternography (NCE-MRC). Nevertheless, false-positive result rates are high with these methods because of viscous secretions and susceptibility artefacts [6, 7]. CE-MRC may be beneficial in equivocal cases [33, 34].

### Intracranial hypotension

Intracranial hypotension is a disease characterised by orthostatic headaches. The cardinal finding is increased intracranial blood volume [35]. Characteristic MRI findings are thickened pachymeninges with contrast enhancement, subdural fluid collections, engorged cerebral veins, rounded dural sinuses, brain sagging, disappearance of the CSF space of the optic nerve sheath and an inferiorly displaced midbrain [35, 36]. These findings are also useful in the follow-up period after treatment. Identifying the presence and location of the CSF leakage is required for a precise diagnosis and accurate management [37]. NCE-MRC obtained with heavily T2W sequences showing the extra-arachnoid fluid may provide additive data by demonstrating meningeal diverticula, high-flow fistulas and/or engorgement of the epidural venous plexus [35]. In equivocal cases, CE-MRC is more sensitive than other methods in demonstrating the leakage location [6].

### Fistulas between the subarachnoid space and inner ear

Although ear trauma-related CSF leakages mostly manifest with otorrhoea, in some patients a fistula between the inner ear and subarachnoid space may develop without associated otorrhoea. This may lead to sensorineural hearing loss (SNHL), which may progress to being persistent unless treated accurately. This condition is a result of posttraumatic fractures that involve the ottic capsule. NCE-MRC demonstrates a decreased fluid level of the inner air. On CE-MRC. intrathecally admistered contrast material is shown to pass into and adjacent to the inner ear [38].

### Virchow-Robin Spaces (VRSs)

If these perivascular spaces filled with CSF reach larger sizes, it may be challenging to differentiate them from other cystic lesions [21, 39]. Heavily T2W sequences (such as 3D-CISS or 3D-SPACE with constant FA mode) are useful in the morphological evaluation of these lesions and their extensions as well as in the differential diagnosis [35, 39].

## Conclusion

MRI is not only beneficial in the diagnosis of CSF-related diseases, but also aids in therapy planning and post-surgery follow-up of the patients. With the advances in MRI systems, newly developed sequences and techniques allow precise evaluation of many CSF-related disorders, the most important of which is hydrocephalus. In order to be able to better assess CSF-related disorders, radiologists should follow new technologies that enable better assessment of CSF hydrodynamics and should apply them in routine use when needed. The PC-MRI and/or 3D-SPACE methods are relatively simple for evaluating true CSF flow and determining the obstruction level. Also, these techniques provide additional physiological information. The 3D-SPACE technique seems to be the most efficient and rapid for evaluating hydrocephalus, ETV and the shunt catheter. In suspicious or complex cases, PC-MRI, 3D-heavily-T2W and/or CE-MRC images may prevent false-negative or -positive results.
